# β- and γ-Actins in the nucleus of human melanoma A375 cells

**DOI:** 10.1007/s00418-015-1349-8

**Published:** 2015-08-04

**Authors:** Marta Migocka-Patrzałek, Aleksandra Makowiecka, Dorota Nowak, Antonina J. Mazur, Wilma A. Hofmann, Maria Malicka-Błaszkiewicz

**Affiliations:** Department of Animal Developmental Biology, Institute of Experimental Biology, Faculty of Biological Sciences, University of Wroclaw, Sienkiewicza 21, 50-335 Wroclaw, Poland; Department of Cell Pathology, Faculty of Biotechnology, University of Wroclaw, Wroclaw, Poland; Department of Physiology and Biophysics, University at Buffalo State University of New York, Buffalo, NY USA

**Keywords:** Actin, Nucleus, Nuclear actin, Actin isoform, Actin polymerization state

## Abstract

Actin is a highly conserved protein that is expressed in all eukaryotic cells and has essential functions in the cytoplasm and the nucleus. Nuclear actin is involved in transcription by all three RNA polymerases, chromatin remodelling, RNA processing, intranuclear transport, nuclear export and in maintenance of the nuclear architecture. The nuclear actin level and polymerization state are important factors regulating nuclear processes such as transcription. Our study shows that, in contrast to the cytoplasm, the majority of endogenous nuclear actin is unpolymerized in human melanoma A375 cells. Most mammalian cells express the two non-muscle β- and γ-actin isoforms that differ in only four amino acids. Despite their sequence similarity, studies analysing the cytoplasmic functions of these isoforms demonstrated that β- and γ-actins show differences in localization and function. However, little is known about the involvement of the individual actin isoforms in nuclear processes. Here, we used the human melanoma A375 cell line to analyse actin isoforms in regard to their nuclear localization. We show that both β- and γ-non-muscle actin isoforms are present in nuclei of these cells. Immunolocalization studies demonstrate that both isoforms co-localize with RNA polymerase II and hnRNP U. However, we observe differences in the ratio of cytoplasmic to nuclear actin distribution between the isoforms. We show that β-actin has a significantly higher nucleus-to-cytoplasm ratio than γ-actin.

**Electronic supplementary material:**

The online version of this article (doi:10.1007/s00418-015-1349-8) contains supplementary material, which is available to authorized users.

## Introduction

Actin is a multifunctional protein that is present in all eukaryotic cells. As a major part of the cytoskeleton, actin is involved in cell motility and maintenance of the eukaryotic cells shape. It takes part in intracellular transport, cytokinesis and signal transduction. Actin is also a constitutive component of the nucleus where it is involved in a number of fundamental nuclear processes. Actin constantly shuttles between cytoplasm and nucleus. The transport rates suggest an active transport mechanism in both directions. Actin is actively imported by importin 9 (Bohnsack et al. 2006; Dopie et al. 2012). In addition, actin contains two nuclear export signals (NES), which help to maintain proper levels of actin in the nucleus (Wada et al. 1998). Actin is exported from the nucleus at least in two ways, through the exportin 1 (Wada et al. 1998) and exportin 6 (Wada et al. 1998; Stüven et al. 2003).

In the cytoplasm, actin is present in monomeric (G-actin) or filamentous (F-actin) form. The equilibrium between the two forms of actin is highly dynamic and strictly controlled (Dominguez and Holmes 2011). In contrast to actin in the cytoplasm, the polymerization state of nuclear actin is still not entirely understood. In some nuclear complexes, actin is present in the form of monomers (Hu et al. 2004; Holaska et al. 2004; Hofmann et al. 2004; Philimonenko et al. 2004; Obrdlik et al. 2008; Simon et al. 2010; Puckelwartz and McNally 2011; Kapoor et al. 2013; Kapoor and Shen 2014). However, actin polymerization is essential for many nuclear processes such as chromatin rearrangement and transcription (Sjölinder et al. 2005; Vieu and Hernandez 2006; Hofmann et al. 2006; Yoo et al. 2007; Dundr et al. 2007; Miyamoto et al. 2011; Obrdlik and Percipalle 2011). A previous study comparing nuclear and cytoplasmic β-actin dynamics showed that nuclear actin can form dynamic oligomers and short polymers (McDonald et al. 2006) reviewed in De Lanerolle and Serebryannyy (2011). Certain conditions such as stress (Munsie and Truant 2012; Domazetovska et al. 2007; Roberts and Baines 2011; Chrustek et al. 2014), serum stimulation (Baarlink et al. 2013) and overexpression of wild-type and mutant actin constructs (Kokai et al. 2014; Kalendová et al. 2014) leads to the appearance of actin filaments in cell nuclei. The equilibrium between monomeric and polymeric actin is important for regulation of gene transcription (Miyamoto and Gurdon 2011). These data strongly suggest that both monomeric and polymeric actins have an important role in cell nucleus. However, observation of nuclear actin polymers, under physiological conditions, is difficult because it cannot be stained by phalloidin—the marker commonly used in filamentous actin (F-actin) staining.

Nuclear actin plays an important role in many nuclear processes. Actin is an essential part of many chromatin remodelling complexes (Olave et al. 2002; Percipalle and Visa 2006; Farrants 2008; Fedorova and Zink 2008), associates with all three RNA polymerases and is necessary for initiation and elongation of transcription (Hu et al. 2004; Fomproix and Percipalle 2004; Hofmann et al. 2004; Philimonenko et al. 2004; Obrdlik et al. 2008). Actin also associates with nascent transcripts (Percipalle et al. 2001, 2002) and is involved in RNA processing and exported by interacting with heterogeneous ribonucleoproteins (Percipalle et al. 2002; Kukalev et al. 2005; Obrdlik et al. 2008; Bogolyubova et al. 2013). Furthermore, actin, together with nuclear myosins, plays an important role in dynamic organization of chromosomal structures in the context of gene expression regulation and genome maintenance (Chuang et al. 2006; Dundr et al. 2007; Hu et al. 2008; Kapoor et al. 2013; Karolczak et al. 2013).

In vertebrates, six actin isoforms are known, two of which, namely β- and γ-non-muscle actin, are expressed in most cells, while the four actin isoforms α-skeletal, α-cardiac, α-smooth muscle and γ-smooth muscle are expressed in a tissue-specific manner (Vandekerckhove and Weber 1978a). The primary structure of actin isoforms is highly conserved, and cytoplasmic β- and γ-actin differ from each other in only four amino acids at their N-terminus (Vandekerckhove and Weber 1978b). Despite their homology, the two cytoplasmic isoforms in fact have different polymerization characteristics (Bergeron et al. 2010) and show spatial segregation in the cytoplasm (Hoock et al. 1991; Le et al. 1998; Popow et al. 2006; Simiczyjew et al. 2014), which suggests that the actin isoforms have distinct biological functions in the cytoplasm (Rubenstein 1990; Khaitlina 2007).

However, there has been no functional study analysing actin isoforms in the nucleus of mammalian cells. We recently confirmed the presence of both non-muscle β- and γ-actin isoforms in the human melanoma A375 cells on protein and the mRNA expression level (Radwanska et al. 2008). We used this cell line to analyse the presence of the non-muscle β- and γ-actin isoforms in the nucleus and its co-localization with two important actin-binding proteins, polymerase RNA II and hnRNP U. In addition, our analysis of the level of endogenous actin polymerization state in the cytoplasm and nucleoplasm of A375 cells showed that majority of nuclear actin is monomeric.

## Results

### Nuclear actin organization

**Fig. 1:**
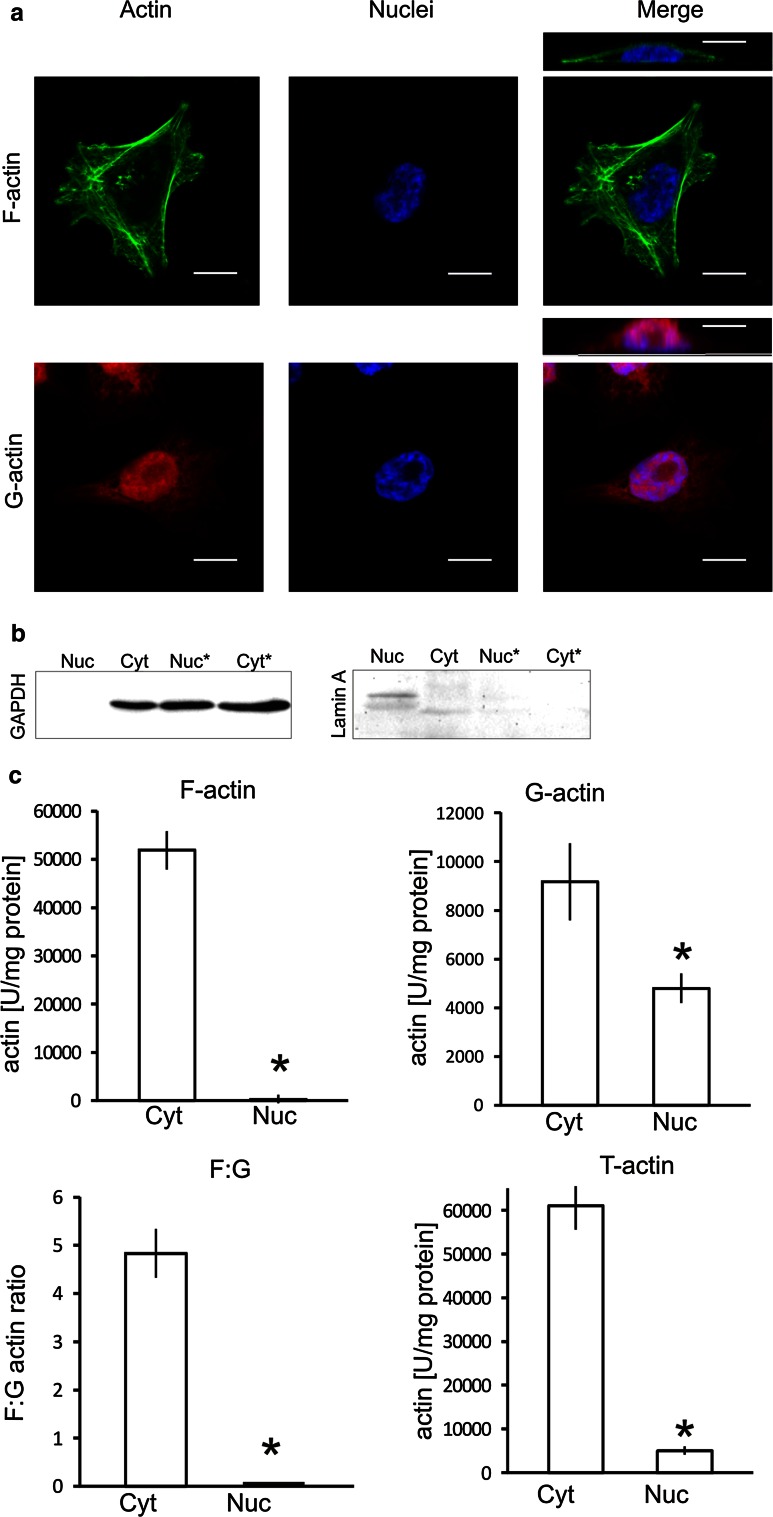
Identification of actin in A375 cells. **a** Confocal microscopy images of monomeric (G) and filamentous (F) actin distribution in A375 cells. DAPI was used to mark the nucleus. Additional, smaller images, shown above merge view, visualize the cross section through the cell. *Scale bar* 150 µm. **b** Immunoblots analysis of nucleoplasm (Nuc) and cytosol (Cyt) purity obtained from A375 cells. Samples were compared with nucleoplasm (Nuc*) and cytosol (Cyt*) obtained using a commercially available kit. Equal amounts of both cellular fractions (50 µg) were separated by SDS-PAGE and probed with antibodies directed against the cytoplasmic protein GAPDH and nuclear protein lamin A. Total protein analysis using Ponceau S staining is shown in supplementary data (Online Resource 2a insert in ‘ESM’). **c** Analysis of actin polymerization state in the cytosol (Cyt) and nucleoplasm (Nuc). *Asterisk* indicates significant differences of value obtained for γ- actin compared to β-actin. The data were obtained from three independent experiments

We started our analysis by visualizing general actin distribution and organization in human melanoma A375 cells by fluorescence microscopy using fluorescently labelled phalloidin that recognizes actin filaments (F-actin) and the Alexa Fluor^®^ 594-labelled DNase I that binds preferentially monomeric actin (G-actin). Confocal images of A375 cells showed, in accordance with previous studies (Hofmann 2009; Percipalle 2013), that phalloidin stained F-actin is exclusively present in the cytoplasm but not in the nucleus. Figure 1a (upper panel) shows that the cytoskeleton in A375 cells is organized in a meshwork of prominent stress fibres in the cell body. In contrast, the Alexa Fluor^®^ 594 DNase I staining that labels monomeric G-actin is mainly seen in the nucleus (Fig. 1a, lower panel).

The nuclear actin polymerization state was confirmed using the method described by Malicka-Blaszkiewicz and Roth (1981) that involves determining the amount of monomeric actin in nuclear and cytoplasmic fractions based on DNase I inhibition.

We confirmed that the nucleoplasm isolation method described by Malicka-Błaszkiewicz (1986, 1990) let us to obtain pure fractions. The absence of cytoplasmic GAPDH from the nucleoplasm clearly demonstrates that this fraction is free of cytoplasmic contaminations. The presence of lamin A, known nuclear protein, in nucleoplasm confirms the proper purification. In contrast, nucleoplasmic fraction obtained using a standard, commercially available kit, contain β-tubulin and no lamin A, indicating cytoplasmic contamination (Fig. 1b).

Monomeric and total actin was measured quantitatively in the cytosol and the nucleoplasm of examined cells by a DNase I inhibition assay under standard conditions. The amount of F-actin and the state of actin polymerization were calculated as described in the Materials and Methods section. The results of this analysis show that in A375 cells, actin in the cytosol is mainly filamentous, while nuclear actin is mostly monomeric (Fig. 1c).

### Identification of β- and γ-actins present in nuclei of A375 cells

**Fig. 2:**
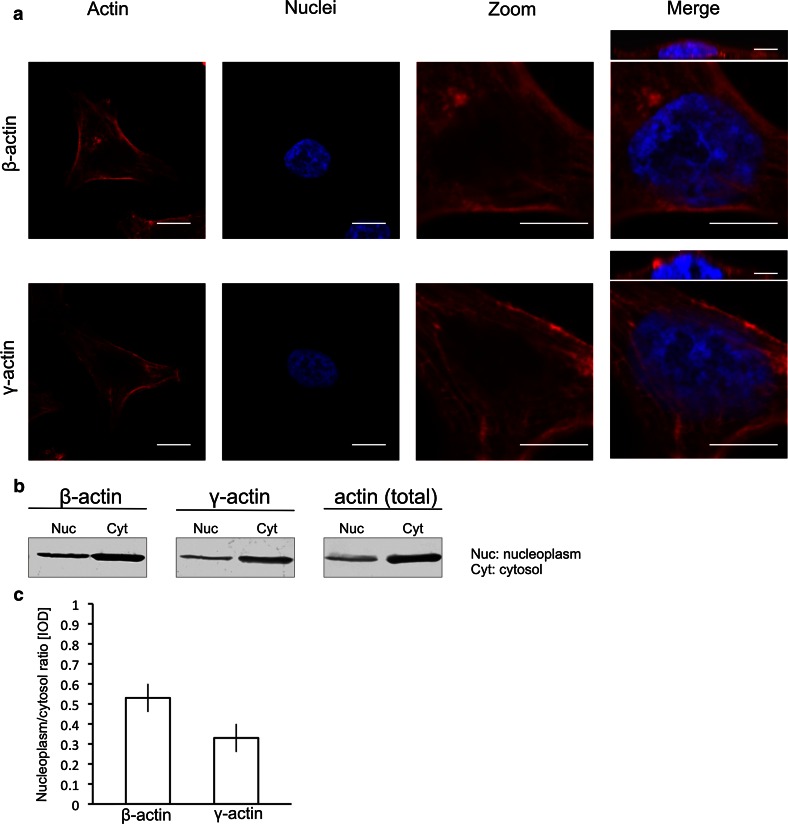
β-and γ- non-muscle actin isoforms identification in cell nuclei. **a** Confocal microscopy images of actin isoforms. A375 cells were fixed and immunostained with either the antibody against β- or γ-actin. DAPI was used to mark the nucleus. Additional, smaller images, shown above merge view, visualize the cross section through the cell. *Scale bar* 150 µm. **b** Immunoblots analysis of actin present in nucleoplasm (Nuc) and cytosol (Cyt) obtained from A375 cells. Equal amounts of each fraction (50 µg) were separated by SDS-PAGE and probed with antibodies directed against β-actin, γ-actin or antibody that recognizes all actin isoforms (total). Total protein analysis using Ponceau S staining is shown in supplementary data (Online Resource 2b insert in ‘ESM’). **c** The integrated optical density (IOD) of the isoform-specific protein bands in nucleoplasm and cytosol was measured, and the nucleoplasm/cytosol ratio of β- and γ-actins was calculated

To determine which actin isoform is present in the nucleus, we stained A375 cells with antibodies that specifically recognize either the β- (Gimona et al 1994) or the γ- (Hanft et al 2006) non-muscle actin isoforms. Immunofluorescence analysis by confocal laser scanning microscopy (Fig. 2a) revealed the presence of β- and γ-actins in the nucleus. The observed low levels of this staining could be due to poor antibody binding. Nuclear actin could be modified, present in different conformation or bound to other proteins, which prevent optimal antibody binding (Steinmetz et al. 1997; Pederson and Aebi 2002; Bettinger et al. 2004; Zhong et al. 2010). The antibody binding to nuclear β- and γ-actins is low even when this isoforms were overexpressed (Online Resource 3 insert in ‘ESM’). However, we confirmed the β- and γ-actins presence in the nucleoplasm and the cytosol, by immunoblotting. Nucleoplasm and cytosol were analysed using two isoform-specific antibodies as well as an antibody that recognizes total actin. As shown in Fig. 2b, both β- and γ-actin isoforms are present in the nucleoplasm and cytosol of A375 cells.

To evaluate the nucleoplasm-to-cytosol ratio of the β- and γ-actins, we measured the protein band density of nucleoplasm and cytosol that were obtained from the immunoblots (Fig. 2b). The two isoforms are expressed in unequal amounts in cells (Khaitlina 2007; Radwanska et al. 2008), and different antibodies could potentially have variations in the binding affinity. For this reason, we did not directly compare the total amount of each isoform in the nucleoplasm. Instead, we determined the nucleoplasm (N)-to-cytosol (C) ratio (N/C) for each isoform independently and compared these. To show the nuclear–cytoplasmic distribution of actin isoforms, we compared the nucleoplasm/cytosol ratio calculated for β- and γ-actins. Our analysis revealed that the nucleus-to-cytoplasm ratio for γ-actin comprises only ~60 % of the ratio for β-actin, i.e. less of the cellular γ-actin is present in the nucleus when compared to β-actin (Fig. 2c).

### Determination of β- and γ-actins co-localization with RNA polymerase II and heterogeneous ribonucleoprotein U

After having determined that β- and γ-actin isoforms are present in the nucleus, we next examined whether both isoforms co-localize with known nuclear actin-binding proteins. Several studies have shown that actin interacts with RNA polymerase II (Philimonenko et al. 2004; Kyselá et al. 2005; Hofmann et al. 2006) and together with heterogeneous ribonucleoprotein U (hnRNP U) is involved in regulation of transcription activation (Kukalev et al. 2005; Bi et al. 2013).

To analyse the co-localization between the β- and γ-actins, RNA polymerase II and hnRNP U, we generated β- and γ-actin constructs that were N-terminally fused to hemagglutinin (HA) protein tag and contained a nuclear localization signal (NLS) between the actin and the protein tag (HA–NLS–β-actin and HA–NLS–γ-actin) to increase the amount of tagged actin in the nucleus for better visualization. N-terminal fusions do not interfere with actin function and have been used successfully to analyse localization and functions of actin in the cytoplasm and the nucleus (Schoenenberger et al. 1999; Posern et al. 2002; McDonald et al. 2006; Chuang et al. 2006; Dundr et al. 2007; Hofmann et al. 2009). HA-tag is small enough to not disrupt intracellular distribution of transfected actin and HA tagged actin can easily enter the nucleus (Müller et al. 2012). NLS-actin constructs have been also used extensively to analyse nuclear actin function (McDonald et al. 2006; Hofmann et al. 2009; Spencer et al. 2011; Baarlink et al. 2013; Kokai et al. 2014; Kalendová et al. 2014). Furthermore, a previous study showed that the increased expression of actin fusion proteins with an artificially added NLS lead to a decrease in endogenous actin level in the nucleus (Hofmann et al. 2009).

**Fig. 3:**
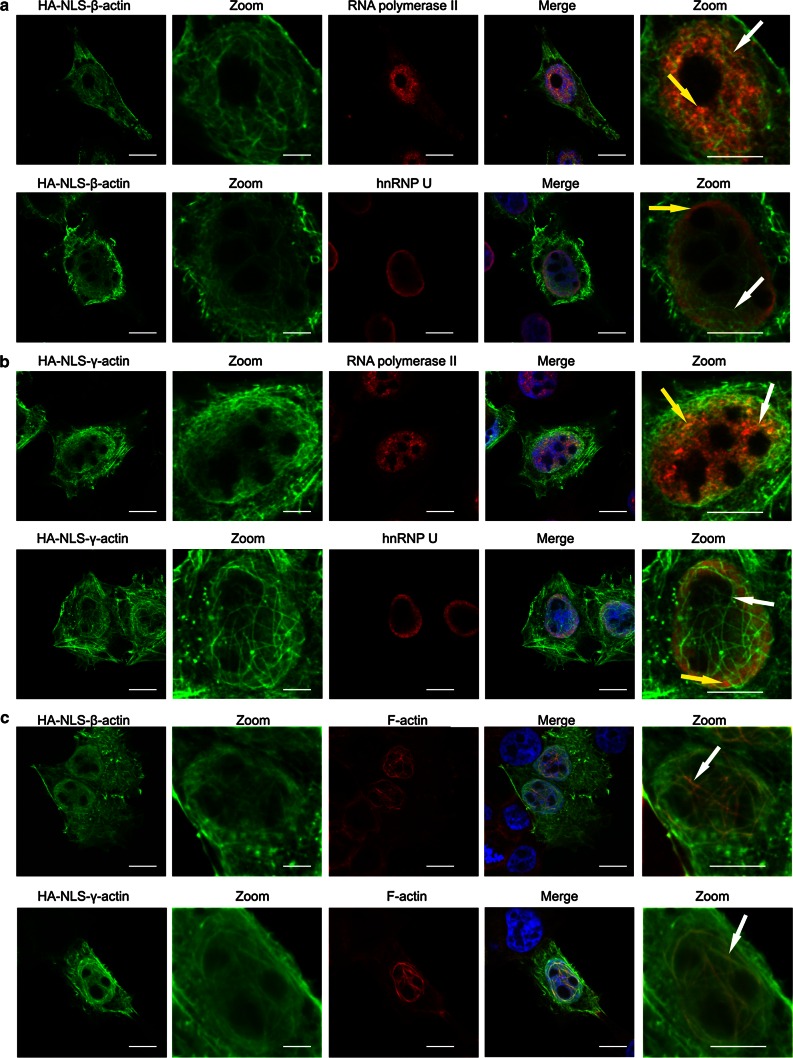
Distribution of β- and γ- non-muscle actin isoforms, filamentous (F) actin, RNA polymerase II and hnRNP U in cell nuclei. **a** Confocal microscopy images of co-localization between β-actin, RNA polymerase II and hnRNP U. Cells were transfected with HA–NLS–β-actin constructs, fixed and stained with anti-RNA polymerase II antibodies (*upper panel*) or anti-hnRNP U antibodies (*lower panel*). **b** Confocal microscopy images of co-localization between γ-actin, RNA polymerase II and hnRNP U. Cells were transfected with HA–NLS–γ-actin construct, fixed and stained with anti-RNA polymerase II antibodies (*upper panel*) or anti-hnRNP U antibodies (*lower panel*). *Scale bar* 150 µm. **c** Confocal microscopy images of β- and γ-actin isoforms and filamentous (F) actin distribution. Cells were transfected with HA–NLS–β-actin (*upper panel*) and HA–NLS–γ-actin (*lower panel*) constructs, were fixed and stained with anti-HA antibody and Alexa Fluor^®^ 546-conjugated phalloidin. *Scale bar* 150 µm. DAPI was used to mark the nucleus. Zoomed images show a high magnification of the nucleus. *White arrows* show examples of bundled filaments of nuclear actin, and *yellow arrows* show examples of co-localization of actin isoforms with RNA polymerase II and hnRNP U

A375 cells were transfected with HA–NLS–β-actin and HA–NLS–γ-actin and, after 24 h, co-immunostained with fluorescently labelled antibodies directed against HA and RNA polymerase II or with antibodies directed against HA and hnRNP U. Confocal microscopy analysis showed that β-actin (Fig. 3a) and γ-actin (Fig. 3b) constructs are dispersed in the nucleoplasm or form actin filaments in the nucleus (white arrows) similarly as described by previous studies (Kokai et al. 2014; Kalendová et al. 2014). Our observations confirm these results not only after β- but also γ-actin overexpression (Fig. 3c). Overexpressed actin isoforms filaments adopt different shapes and form meshwork in the whole nucleus, except nucleoli. β-Actin and γ-actin constructs co-localize both with RNA polymerase II (yellow arrows, upper panels) and with hnRNP U (yellow arrows, lower panels). This observed co-localization was confirmed by calculation of the overlap coefficient, a method to measure the degree of co-localization of objects in confocal dual-colour images (Zinchuk et al. 2005). Values range from 0 to 1 (0: no co-localization, 1: all pixels co-localize). The average overlap coefficient value for β-actin with both RNA polymerase II and hnRNP U is 0.95. The average overlap coefficient value for γ-actin with RNA polymerase II is 0.92 and for hnRNP U 0.95. The fluorescence images and the calculated co-localization values suggest that both isoforms not only localize to the nucleus but also show similar colocalization pattern with important nuclear proteins.

To control whether HA-tag, used to detect actin isoforms, does not enter the nucleus itself, we compare non-transfected A375 cells (Online Resource 1a insert in ‘ESM’) with cells transfected with construct containing only HA (Online Resource 1b insert in ‘ESM’). HA does not enter to the nucleus itself and does not change intracellular distribution of RNA polymerase II and hnRNP U.

## Discussion

In recent years, the presence of actin in the nucleus as well as its physiological significance has been indisputably demonstrated (Pederson and Aebi 2005; Migocka-Patrzałek and Malicka-Błaszkiewicz 2009; Hofmann 2009; Visa and Percipalle 2010; Skarp and Vartiainen 2010). An increasing body of evidence shows that the nuclear actin level and its polymerization state are strictly controlled. However, the polymerization state of actin remains an open question.

Similar to other previous studies (Percipalle 2013), we did not observe F-actin in the cell nucleus using phalloidin conjugated with a fluorophore and microscopy methods. However, the monomeric actin, detected with Alexa Fluor^®^ 594 labelled DNase I, was clearly visible in the cell nucleus. These results were corroborated using actin polymerization assay, measuring the actin content as a DNase I inhibitor—the only method so far measuring quantitatively endogenous actin level and polymerization state directly in biological fractions such as cytoplasm or nucleoplasm. Our results show that the endogenous nuclear actin is mainly monomeric under test conditions. In contrast, we detect F-actin in cells overexpressing β- and γ-actins. This observation suggests that conditions in the nucleus, such as ions, ATP and nucleation factors concentration, are appropriate for actin filaments formation.

An important aspect of actin regulation, which has not been yet analysed in detail, is the existence of β- and γ-actins in the nucleus. We report here that nuclear actin is not comprised of a single actin isoform but that the nuclear actin pool consists of β- and γ-actins. The difficulties in visualization of β- and γ-actins in the cell nucleus using microscopic techniques may be due to actin modifications, binding to other proteins or different conformation (Steinmetz et al. 1997; Pederson and Aebi 2002; Bettinger et al. 2004; Zhong et al. 2010). However, we confirmed the presence of β- and γ-actin isoforms in the nucleoplasm, as well as in the cytosol, by immunoblotting. The nucleus-to-cytoplasm ratio is higher for β- than for γ-actin suggesting that the nuclear level of each actin isoform is controlled independently. Different actin isoforms were observed also in other kind of cells, such as primary mouse embryonic fibroblasts (Tondeleir et al. 2012) suggesting that the presence of various actin isoforms is a conserved phenomenon.

The presence of β- and γ-actin isoforms could have important implications for our understanding of nuclear actin function. Even though these two isoforms differ by only four amino acids (Vandekerckhove and Weber 1978b), they show differences in their biochemical and physical properties, and have distinct physiological functions. A recent study showed that β- and γ-non-muscle actins differ in their polymerization and depolymerization kinetics. The γ-actin has a much slower rate of polymerization but forms more stable polymers. In contrast, β-actin exhibits a more dynamic behaviour with faster nucleotide exchange rates (Bergeron et al. 2010).

In this context, the observed differences in nucleus-to-cytoplasm ratio between the β- and γ-actin isoforms are of interest. In most cells, non-muscle β- and γ-actin isoforms are expressed at ratio of 2:1, and changes in this ratio are usually associated with pathological processes such as metastatic potential of cancer cells (Le et al. 1998; Popow et al. 2006; Radwanska et al. 2008). Interestingly, a recent study analysing nuclear β-actin level in response to growth regulation suggests that changes in cellular environment also affect the nuclear-to-cytoplasmic ratio of β-actin (Spencer et al. 2011). The study showed that induction of cell quiescence leads to changes in the nuclear-to-cytoplasm actin ratio, specifically to decrease nuclear β-actin level. Actin polymerization is crucial for the transcription transition from initiation to elongation and to maintain the latter process (Percipalle 2013, 2014). In the light of the discovery that β-actin, in comparison with γ-actin, shows a more dynamic kinetic behaviour (Bergeron et al. 2010), together with recent findings that β-actin is the isoform necessary in gene expression regulation (Tondeleir et al. 2012), one could speculate that quiescent cells with reduced transcriptional activity require less of this dynamic actin isoform.

Our results show that both actin isoforms, β and γ, co-localize with RNA polymerase II and hnRNP U. These data suggest that both isoforms may be important in nuclear processes. Different nuclear levels of each actin isoform in the nucleus may be a way to control different parameters such as the speed or rate of actin polymerization. However, detailed studies on the distribution of both isoforms in the nucleus under different physiological conditions are required to form a valid hypothesis.

The notion that β- and γ-actin isoforms might have individual nuclear functions is supported by a study analysing plant actin variants in nuclei of *Arabidopsis thaliana* (Kandasamy et al. 2010). The authors show that different actin variants show preferential localization to nuclear substructures such as speckles or nucleoli. We have not observed specific spatial segregations of the human nuclear β- and γ-actin isoforms in our analysis. However, the analysed plant actin subclasses show, with 7 % divergence, a much greater difference in amino acid composition compared to the less than 1 % divergence between the mammalian non-muscle β- and γ-actins, which could explain more subtle differences in nuclear localization that we observed.

At this point, we can only speculate about the physiological significance of the presence of β- and γ-actins in the nucleus. Actin is involved in many different steps of transcription regulation (Hofmann 2009; Visa and Percipalle 2010; Percipalle 2013). So far it is not clear how these different functions of actin are regulated. One of the most pertinent questions in this regard concerns the polymerization state of nuclear actin. Our results show that the endogenous nuclear actin is mainly monomeric. A potential mechanism of actin polymerization regulation could be the involvement of different actin isoforms that exhibit differences in polymerization kinetics (Bergeron et al. 2010) and thus dynamic behaviour depending on the requirements of the nuclear processes.

In summary, we present evidence that the nuclear actin pool comprises β- and γ-actins. While the cause and physiological significance of the presence of β- and γ-actins in the nucleus has yet to be determined, based on the observed differences in nuclear-to-cytoplasmic ratio in combination with the unique physical and biochemical properties, we suggest that the various actin isoforms may have specific nuclear functions. Discerning the individual roles of these isoforms in the nucleus might lead to a better understanding of nuclear actin functions.

## Materials and methods

### Cell culture and media

Human melanoma A375 cells were obtained from the American Type Culture Collection (Manassas, VA, USA). Cells were cultivated in Dulbecco’s modified Eagle’s medium, supplemented with foetal bovine serum to a final concentration of 10 % and antibiotics (10 U/ml penicillin and 10 µg/ml streptomycin), at 37 °C under a humidified atmosphere of 5 % CO_2_.

### Antibodies and fluorescent markers

Mouse monoclonal antibodies directed against all actin isoforms (clone AC40), mouse monoclonal β-actin (clone AC-15) (Gimona et al. 1994) and γ-actin (clone 2-2.1.14.17) (Hanft et al. 2006) were obtained from Sigma-Aldrich (St Louis, MO, USA). Alexa Fluor^®^ 546-conjugated phalloidin, used to visualize actin filaments, and Alexa Fluor^®^ 594-labelled DNase I binding monomeric actin, were obtained from Invitrogen (Waltham, MA, USA). Mouse monoclonal antibodies directed against RNA polymerase II (clone 8WG16) were obtained from Covance (Princeton, NJ, USA), and hnRNP U and GAPDH were from Santa Cruz Biotechnology (Santa Cruz, CA, USA). Goat polyclonal antibodies directed against HA were obtained from Santa Cruz Biotechnology (Santa Crus, CA, USA). Nucleic acid staining reagent DAPI and rabbit polyclonal antibodies directed against lamin A were obtained from Sigma-Aldrich (St Louis, MO, USA). Dako cytomatic fluorescent mounting medium was obtained from Dako (Glostrup, Denmark). For immunocytochemistry studies, the donkey anti-mouse labelled with CyTM2, obtained from Jackson ImmunoResearch Laboratories (West Grove, PA, USA), the rabbit anti-mouse labelled with TRITC or FITC, and donkey anti-goat labelled with FITC, obtained from Sigma-Aldrich (St Louis, MO, USA), secondary antibodies, were used. For immunoblot analysis, the donkey anti-mouse secondary antibodies conjugated with horseradish peroxidase were obtained from Jackson ImmunoResearch Laboratories (West Grove, PA, USA).

### Actin polymerization state

Actin was determined as inhibitor of DNase I from bovine pancreas (Sigma-Aldrich, St Louis, MO, USA) under standard assay conditions (Malicka-Blaszkiewicz and Roth 1981). The amount of monomeric actin (G-actin) was measured by DNase I inhibition, directly in the cytosol and nucleoplasm obtained from A375 cells. Total actin (T-actin) content was measured after dilution of the samples, using G-actin stabilizing buffer A (10 mM Tris–HCl pH 7.4, 1 mM dithiothreitol, 0.1 mM ATP, 0.1 mM CaCl_2_, 0.25 M sucrose). For the measurement of maximal inhibition, a specific dilution below the critical actin concentration had to be applied to completely depolymerize the F-actin. The amount of F-actin was calculated by subtracting the amount of G-actin from the total actin (F = T − G). The state of actin polymerization was defined by the F-to-G-actin ratio (F:G). One unit of DNase I inhibitor (actin) is the amount that reduces the activity of 20 ng of DNase I by 10 % under standard assay conditions (Malicka-Blaszkiewicz and Roth 1981; Malicka-Blaszkiewicz 1986). Actin concentration was expressed in units of DNase I inhibitor per milligram of sample protein. The experiments were performed independently three times. Each experiment consisted of three measurements.

### Plasmids and cell transfection

The following expression constructs were used in this study: p3xHA-C1–NLS-β-actin (HA–NLS–β-actin), p3xHA-C1–NLS-γ-actin (HA–NLS–γ-actin) and empty plasmid p3xHA-C1 (Müller et al. 2012). Constructs were generated in two steps. The cDNA encoding cytoplasmic human β- or γ-actin, respectively, with their 3′UTRs (untranslated regions) was cloned into pOctA-C1–NLS plasmid. pOCTA-C1–NLS plasmid was generated by replacing the sequence coding for EGFP from pEGFP-C1 plasmid (Takara Bio, Otsu, Japan) with the sequence coding for OctA-tag with NLS. The primers used for amplifying the β- or γ-actin isoform cDNAs were as follows: β-actin forward 5′ G ATG GAT GAT GAT ATC GCC GCG 3′ and reverse 5′ G CTA AGG TGT GCA CTT TTA TTC AAC 3′; γ-actin forward 5′ G ATG GAA GAA GAG ATC GCC GC 3′ and reverse 5′ G GG TTA CGG CAG CAC TTT TAT TTT 3′. PCR inserts were cloned into pOctA-C1–NLS using *Xho*I restriction sites. Obtained cDNA for β- or γ-actin with additional NLS sequence were cloned into p3xHA-C1 plasmid. Primers used for cloning β-actin or γ-actin isoform with NLS sequence from pOCTA-C1–NLS-ACTB 3′UTR vector into p3xHA-C1: NLS–β-actin forward 5’ GTT CCT AAG AAG AAG CGT AAG 3′ and 5′ G CTA AGG TGT GCA CTT TTA TTC AAC 3′; NLS- γ-actin forward 5′ GTT CCT AAG AAG AAG CGT AAG 3′ and 5′ G GG TTA CGG CAG CAC TTT TAT TTT 3′. PCR inserts were cloned into p3xHA-C1 using *Xho*I and *Xba*I restriction sites.

Lipofectamine™ 2000 (Thermo Fisher Scientific, Waltham, MA, USA), a liposomal transfection reagent, was used to transfect A375 cells with the empty p3xHA-C1 plasmid or the p3xHA-C1 plasmids encoding human β- or γ-actin with or without NLS according to the manufacturer’s protocol. Twenty-four hours after transfection, cells were used for further experiments.

### Microscopic techniques

A375 cells, grown on glass coverslips, were transfected with the indicated constructs. Twenty-four hours after transfection, cells were fixed with 4 % paraformaldehyde in phosphate-buffered saline (PBS) for 20 min. Coverslips were mounted with Prolong antifade containing 4′,6′-diamino-2-phenylindole (Invitrogen, Carlsbad, CA, USA) and analysed with a confocal laser scanning microscopy (Olympus FLUOVIEW FV1000). The optical section at the microscope was always focused on the nucleus level. For immunofluorescence staining, cells were permeabilized with 0.1 % Triton X-100 in PBS for 10 min after fixation, washed three times with PBS and incubated with 1 % bovine serum albumin (BSA) in PBS for 1 h, followed by incubation with appropriate primary and fluorescently labelled secondary antibodies. Cells were mounted as described above followed by microscopic analysis. Images were processed using Corel or ImageJ (NIH) software.

Co-localization of proteins was determined using the overlap coefficient (Zinchuk et al. 2005), a method to measure the degree of co-localization of objects in confocal dual-colour images. Overlap coefficient after Manders and Verbeek (1993) is relative number of co-localizing pixels, as compared to the total number of pixels above threshold. It was calculated using Olympus software. Values range from 0 to 1 (0: no co-localization, 1: all pixels co-localize). Co-localization analysis results were calculated as an average value measured in 12 cells.

### Isolation of cytosol

Cells were homogenized and cytosol was obtained using the method described by Malicka-Blaszkiewicz and Roth (1981). The A375 cells, grown in cell culture dishes, were gently washed with PBS, scraped with a rubber policeman and suspended in freshly made G-actin stabilizing buffer A (10 mM Tris–HCl pH 7.4, 1 mM dithiothreitol, 0.1 mM ATP, 0.1 mM CaCl_2_, 0.25 M sucrose). Cells were centrifuged (1000×*g*, 3 min, 4 °C) and homogenized with three volumes of buffer A using Dounce homogenizer. Homogenates were centrifuged at high speed (105,000×*g*, 1 h, 4 °C). Supernatant was used as the cytosol and stored at −70 °C for further experiments.

### Isolation of cells nuclei

Cells were homogenized and cell nuclei were isolated using the method described by Wallace et al. (1977). The A375 cells, grown in cell culture dishes, were gently washed with PBS, scraped with a rubber policeman and suspended in buffer B (10 mM Tris–HCl pH 7.4, 0.25 M sucrose, 6 mM KCl, 5 mM magnesium acetate, 0.1 mM EGTA). Cells were centrifuged (1000×*g*, 3 min, 4 °C) and homogenized with three volumes of buffer B using Dounce homogenizer. Homogenates were suspended in ten volumes of buffer B and centrifuged (1000×*g*, 10 min, 4 °C). The pellets were gently washed twice with ten volumes of buffer B containing 0.5 % Nonidet-P40 and twice in ten volumes of buffer B containing 0.5 % Nonidet-P40 and 0.1 mM PMSF. Each time samples were washed around 100 times with glass Pasteur pipettes with long tip and centrifuged at the same conditions (1000×*g*, 10 min, 4 °C). Pellets were washed for the last time in ten volumes of buffer B and centrifuged at 25,000×*g*, for 20 min, at 4 °C. Pellets containing the cells nuclei were immediately used to isolate nucleoplasm.

### Isolation of nucleoplasm

Nucleoplasm was isolated according to Malicka-Blaszkiewicz (1986). Cell nuclei were resuspended in buffer A, centrifuged (1000×*g*, 10 min, 4 °C) and opened by gentle homogenization with three volumes of buffer A in Dounce homogenizer. Homogenates were centrifuged at high speed (105,000×*g*, 1 h, 4 °C). Supernatant was used as nucleoplasm and stored at −70 °C for further experiments.

### Isolation of cytosol and nucleoplasm using commercially available kit

A375 cells grown in cell culture dishes were utilized to obtain cytosol and nucleoplasm using NucBuster™ Protein Extraction Kit Merck (Darmstadt, Germany) according to manufacturer instructions.

### Immunoblot analysis

A375 cells were grown under standard conditions. Twenty-four hours after seeding, nucleoplasm and cytosol were prepared. The protein concentration was determined (Bradford 1976), and identical amounts of proteins (50 μg) were separated by sodium dodecyl sulphate–polyacrylamide gel electrophoresis (SDS-PAGE) according to Laemmli (1970) and transferred to nitrocellulose membranes (Towbin et al. 1979). After transfer, total protein analysis after Ponceau S staining (Sigma-Aldrich, St Louis, MO, USA) was applied as a loading control (Online Resource 2 insert in ‘ESM’). Ponceau S staining is considered to be the best method for total protein analysis during cellular different fractions comparison (Eaton et al. 2013; Dittmer and Dittmer 2006). The membrane staining with Ponceau S shows, however, the different colour intensity of analysed fractions (cytosol and nucleoplasm). Membrane-associated proteins, which are highly hydrophobic, aggregate during boiling samples at 95 °C for 10 min (Schägger 2006). Such aggregates stopped in the stacking gel and are absent in the separating gel and finally on the membrane. Also small proteins (below 10 kDa) could be not present on the membrane (Schägger 2006). The blots were probed with the indicated specific antibodies. The immunoreactive bands were detected by enhanced chemiluminescence using Western Lightning Plus-ECL kit obtained from PerkinElmer (Waltham, MA, USA) according to manufacturer instructions. The intensity (pixels) of staining of the bands interacting with the proper antibodies was analysed using ImageJ (NIH) software.

### Statistical analysis

All data are given as means ± standard deviations, and their significance was determined with Student’s *t* test. The significance level was set at *P* < 0.05.

## Electronic supplementary material

Supplementary material 1 (PDF 177 kb)

Supplementary material 2 (PDF 171 kb)

Supplementary material 3 (PDF 174 kb)
