# Supplementary material for: β- and γ-Actins in the nucleus of human melanoma A375 cells
Source: Histochem Cell Biol. 2015 Aug 4;144(5):417–28. doi: 10.1007/s00418-015-1349-8 (PMC4628621; doi:10.1007/s00418-015-1349-8)
Supplement: Supplementary file 1 — Supplementary material 1 (PDF 177 kb) [file 418_2015_1349_MOESM1_ESM.pdf]

β- and γ-Actin in the Nucleus of Human Melanoma A375 Cells

Histochemistry and Cell Biology,

Marta Migocka-Patrzałek[1,2,3*], Aleksandra Makowiecka[2], Dorota Nowak[2], Antonina J. Mazur[2], Wilma A. Hofmann[3], Maria Malicka-Błaszkiewicz[2]

*Corresponding author:

Marta Migocka-Patrzałek

Department of Animal Developmental Biology,

Institute of Experimental Biology,

Faculty of Biological Sciences,

University of Wroclaw,

Sienkiewicza 21,

50-335 Wroclaw, Poland

Tel: 0048 71 375 40 23

Fax: 0048 71 375 28 95

e-mail: marta.migocka-patrzalek@uni.wroc.pl

ESM.1 Transfection control

a Confocal microscopy images of non-transfected A375 cells. Cells were fixed and immunostained with antibodies against hemagglutinin (HA), RNA polymerase II and hnRNP U.

b Confocal microscopy images of A375 cells transfected with construct containing only HA. Cells were fixed and immunostained with antibodies against HA, RNA polymerase II and hnRNP U. DAPI was used to mark the nucleus. Scale bar 150 µm.

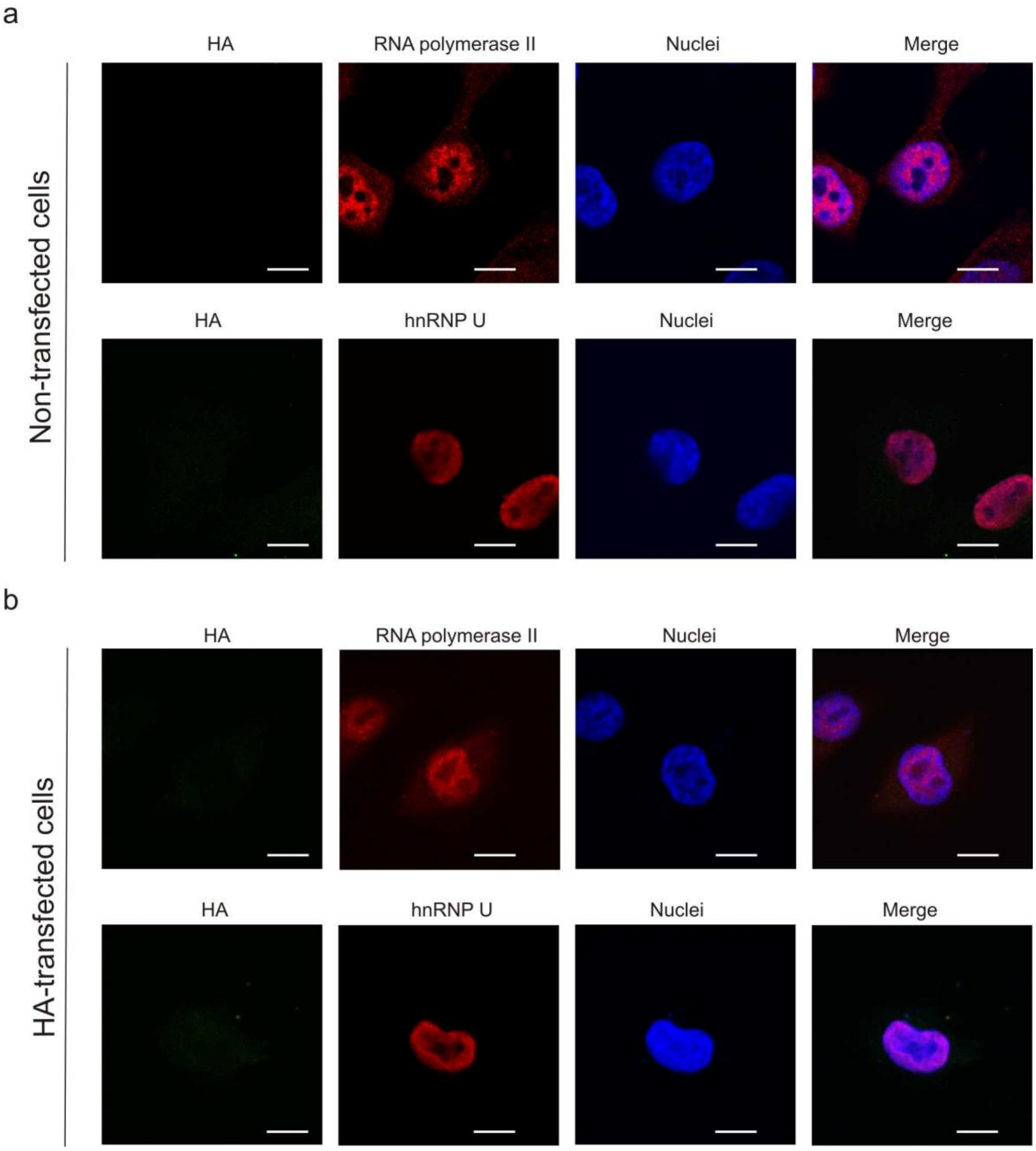
a
HA
RNA polymerase II
Nuclei
Merge
Non-transfected cells
HA
hnRNP U
Nuclei
Merge
b
HA
RNA polymerase II
Nuclei
Merge
HA-transfected cells
HA
hnRNP U
Nuclei
Merge
