# Supplementary material for: β- and γ-Actins in the nucleus of human melanoma A375 cells
Source: Histochem Cell Biol. 2015 Aug 4;144(5):417–28. doi: 10.1007/s00418-015-1349-8 (PMC4628621; doi:10.1007/s00418-015-1349-8)
Supplement: Supplementary file 2 — Supplementary material 2 (PDF 171 kb) [file 418_2015_1349_MOESM2_ESM.pdf]

β- and γ-Actin in the Nucleus of Human Melanoma A375 Cells

Histochemistry and Cell Biology,

Marta Migocka-Patrzałek[1,2,3*], Aleksandra Makowiecka[2], Dorota Nowak[2], Antonina J. Mazur[2], Wilma A. Hofmann[3], Maria Malicka-Błaszkiewicz[2]

*Corresponding author:

Marta Migocka-Patrzałek

Department of Animal Developmental Biology,

Institute of Experimental Biology,

Faculty of Biological Sciences,

University of Wroclaw,

Sienkiewicza 21,

50-335 Wroclaw, Poland

Tel: 0048 71 375 40 23

Fax: 0048 71 375 28 95

e-mail: marta.migocka-patrzalek@uni.wroc.pl

ESM.2 Total protein analysis

Equal amounts (50 µg) of fractions were separated in SDS electrophoresis, transferred to membranes and stained using Ponceau S solution.

a Total protein staining for immunoblot analysis of nucleoplasm (Nuc) and cytosol (Cyt) purity, compared with nucleoplasm (Nuc*) and cytosol (Cyt*) obtained using a commercially available kit (showed in Fig. 1b). Left membrane was further probed with antibodies directed against the GAPDH, right against lamin A.

b Total protein staining for immunoblot analysis of actin present in nucleoplasm and cytosol (showed in Fig. 2b). Molecular weight (MW) is given in kDa.

a

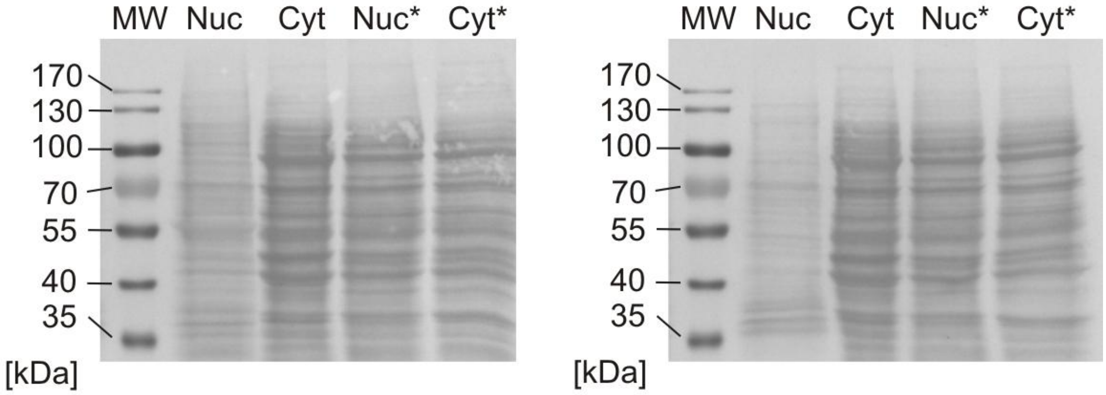

MW Nuc Cyt Nuc* Cyt*
170
130
100
70
55
40
35
[kDa]
MW Nuc Cyt Nuc* Cyt*
170
130
100
70
55
40
35
[kDa]

b

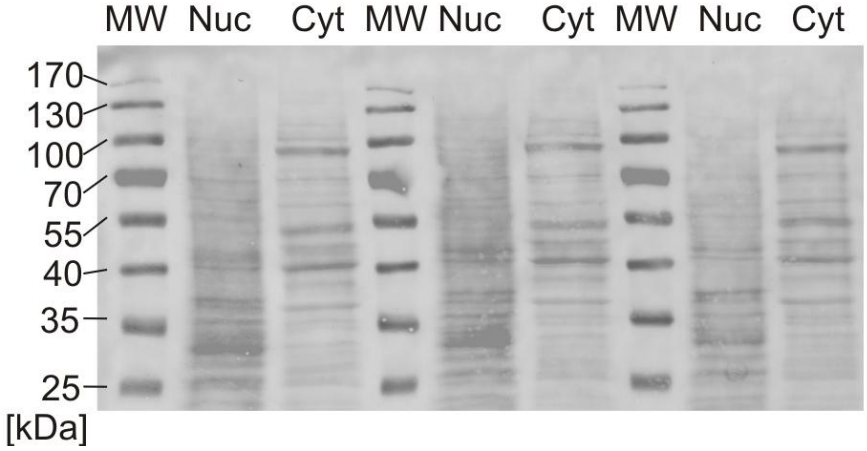

MW Nuc Cyt MW Nuc Cyt MW Nuc Cyt
170
130
100
70
55
40
35
25
[kDa]
