# Supplementary material for: β- and γ-Actins in the nucleus of human melanoma A375 cells
Source: Histochem Cell Biol. 2015 Aug 4;144(5):417–28. doi: 10.1007/s00418-015-1349-8 (PMC4628621; doi:10.1007/s00418-015-1349-8)
Supplement: Supplementary file 3 — Supplementary material 3 (PDF 174 kb) [file 418_2015_1349_MOESM3_ESM.pdf]

β- and γ-Actin in the Nucleus of Human Melanoma A375 Cells

Histochemistry and Cell Biology,

Marta Migocka-Patrzałek[1,2,3*], Aleksandra Makowiecka[2], Dorota Nowak[2], Antonina J. Mazur[2], Wilma A. Hofmann[3], Maria Malicka-Błaszkiewicz[2]

*Corresponding author:

Marta Migocka-Patrzałek

Department of Animal Developmental Biology,

Institute of Experimental Biology,

Faculty of Biological Sciences,

University of Wroclaw,

Sienkiewicza 21,

50-335 Wroclaw, Poland

Tel: 0048 71 375 40 23

Fax: 0048 71 375 28 95

e-mail: marta.migocka-patrzalek@uni.wroc.pl

ESM.3 Identification of actin in A375 cells overexpressing β- and γ-actin

a Confocal microscopy images of A375 cells transfected with HA-NLS-β-actin construct. Cells were fixed and immunostained with antibodies against HA and γ-actin.

b Confocal microscopy images of A375 cells transfected with HA-NLS-γ-actin construct. Cells were fixed and immunostained with antibodies against HA and β-actin. DAPI was used to mark the nucleus. Scale bar 150 μm.

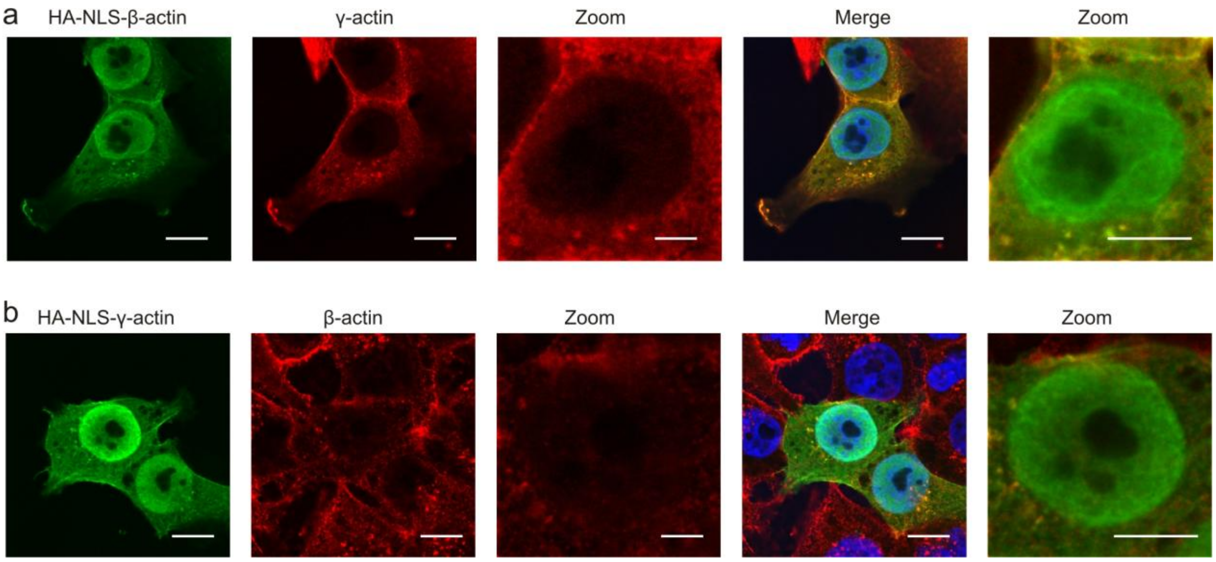
a
HA-NLS-β-actin
γ-actin
Zoom
Merge
Zoom
b
HA-NLS-γ-actin
β-actin
Zoom
Merge
Zoom
